# Pulmonary Function in HIV-Infected Children at a Tertiary Care Hospital in North India: A Prospective Cross-Sectional Study

**DOI:** 10.7759/cureus.46935

**Published:** 2023-10-13

**Authors:** Priyanka Gupta, Naresh Kumar

**Affiliations:** 1 Pulmonary Medicine, Lifecare Hospital, Burjeel Holdings, Abu Dhabi, ARE; 2 Pulmonary Medicine, Sawai Man Singh (SMS) Hospital, Jaipur, IND

**Keywords:** viral load, cd4 count, spirometry, hiv-infected children, pulmonary function

## Abstract

Background

The global burden of HIV remains significant, particularly in India. Antiretroviral therapy (ART) has improved outcomes for children with HIV, yet understanding the virus's impact on respiratory health is essential. Pulmonary complications, common in HIV-infected adults, are poorly understood in children. Despite India's high HIV prevalence, data on pediatric lung function are lacking. This study aims to evaluate spirometry-based pulmonary function in perinatally HIV-infected children, exploring associations with disease severity, immune status, and other factors.

Methods

This prospective cross-sectional study conducted in a North Indian tertiary care hospital aimed to assess pulmonary function using spirometry in children (6-18 years) with HIV infection. Ethical approval and informed consent were secured. Data on demographics, clinical history, CD4+ T-cell counts, and viral load were collected. Certified respiratory therapists performed spirometry using standardized protocols. Descriptive statistics were computed, and differences in pulmonary function based on CD4+ T-cell counts, viral load, and opportunistic infection were analyzed. The study adhered to ethical guidelines and maintained participants' confidentiality.

Results

This cross-sectional study enrolled 57 children (mean age 13.6±3.2 years) with HIV infection. Age distribution was <9 years (24.6%), 9-11 years (28.1%), and >11 years (47.4%). Males constituted 56.1%. The mean BMI was 15.92±2.78 kg/m². HIV viral load (87.23±56.28 copies/μL) and CD4 count (1146.32±103.98 cells/mm³) were recorded. ART duration averaged 6.21±1.36 years. Viral load groups were <1 (52.6%), 1-1000 (26.3%), and >1000 copies/μL (21.1%). CD4 categories were >500 cells/mm³ (47.4%), 200-499 (42.1%), and <200 cells/mm³ (10.5%). Spirometry showed 71.9% normal and 28.1% abnormal (mild/moderate obstruction: 18.8%, mild/moderate restriction: 81.3%). No significant spirometric differences were observed among CD4 or viral load groups (p>0.05), nor with opportunistic infections (p>0.05).

Conclusion

This study reveals complex associations between spirometric parameters and CD4 count, viral load, and opportunistic infections in children with HIV. Further research, including longitudinal studies, is needed to unravel the intricate interplay and improve management strategies for this population.

## Introduction

HIV remains a significant global health challenge, particularly in India, where transmission of the virus continues to be a major concern [1]. The introduction of antiretroviral therapy (ART) has significantly improved the prognosis and quality of life for children with HIV infection. However, despite these advancements, the long-term consequences of HIV infection, including its impact on respiratory health, continue to warrant investigation [2]. Notably, India ranks third globally in terms of its population grappling with HIV infection. The National Association of Counties reports that approximately 0.70 lakh (0.54-0.89 lakh) children are living with HIV infections in India during the year 2021 [3].

Pulmonary complications are recognized as a common co-morbidity in HIV-infected individuals, especially in adults living with the virus. Recent research has shown that pulmonary complications are observed in a notable proportion of adults who are HIV positive, ranging from 31% to 64% [4,5]. Among these individuals, spirometry assessments have indicated up to 20% prevalence of airway obstruction, while clinically diagnosed asthma has been identified in around 20% of cases [4,5]. Studies have revealed a higher prevalence of respiratory disorders, such as pneumonia, bronchitis, and tuberculosis, in adults with HIV [6,7]. Moreover, the immune dysregulation caused by HIV infection can lead to chronic inflammation and lung damage, contributing to respiratory impairment. However, while the effects of HIV on adult lung health have been relatively well-studied, our understanding of its impact on the developing respiratory system in children with perinatal HIV infection remains limited [8].

The early years of life represent a critical period for lung development, and any insult during this stage may have long-lasting consequences on pulmonary function [9]. Children with HIV infection are exposed to various risk factors, including prenatal exposure to HIV itself, opportunistic infections, and ART-related toxicities, all of which could potentially affect lung growth and function [10]. Respiratory illness stands as the predominant expression of HIV/AIDS in children, constituting over 50% of HIV-related fatalities [11]. Investigating the pulmonary function of these children is of paramount importance to comprehend the extent of respiratory compromise and develop targeted interventions to improve their overall health outcomes [12,13].

The present study aimed to evaluate pulmonary function using spirometry in children with perinatal HIV infection. By employing spirometric measurements, we seek to identify patterns of lung impairment, investigate potential associations with disease severity, and explore the impact of various factors, including CD4 count and opportunistic infections, on the respiratory health of these children.

## Materials and methods

Study design and participants

This prospective cross-sectional study was conducted under the department of pediatrics and pulmonary medicine at SGK Hospital, Jaipur, involving children with HIV infection for a period of one year (March 2018 to March 2019). The study enrolled children between the ages of 6 and 18 years who had a confirmed diagnosis of HIV infection based on medical records and laboratory tests. Children with a history of chronic lung diseases unrelated to HIV, congenital heart diseases, or any other condition affecting lung function were excluded from the study. From January 2013 to December 2017, a total of 225 children with HIV attended pediatric outpatient/inpatient. A sample size of 57 was calculated considering the proportion of children with HIV having an abnormal pulmonary function as 18% with a CI of 95% and a margin of error of 10% [9]. Thus, using a computer-generated random number, 57 children were selected randomly from 225 children.

Data collection

Data was collected using predesigned proforma. Demographic and clinical information, including age, gender, duration of HIV diagnosis, ART history, history of opportunistic infections, and co-morbidities, were recorded for each participant. Additionally, information regarding the CD4+ T-cell counts and viral load at the time of enrolment was recorded.

Spirometry assessment

Pulmonary function testing (PFT) was conducted by certified respiratory therapists using a standardized spirometry protocol. Participants were advised to refrain from eating a heavy meal, engaging in strenuous physical activity, or using bronchodilators for at least two hours prior to the spirometry assessment. Each participant underwent spirometry in a quiet, controlled environment to minimize confounding factors. Spirometry was performed using a digital spirometer (SP10: Contec Medical Systems CO., LTD, China], which meets the American Thoracic Society (ATS)/European Respiratory Society (ERS) standards for accuracy and reproducibility. Each participant was instructed on the proper technique, and three acceptable efforts were obtained. The best effort meeting the ATS/ERS acceptability and repeatability criteria was recorded for analysis.

The spirometric parameters recorded included forced vital capacity (FVC), the maximum volume of air exhaled forcefully after a deep inhalation; forced expiratory volume in one second (FEV1), the volume of air expelled during the first second of forced exhalation; FEV1/FVC ratio, the ratio of FEV1 to FVC, representing the proportion of exhaled air in the first-second relative to the total exhaled volume; and peak expiratory flow rate (PEFR), the maximum flow rate achieved during forceful exhalation.

Statistical analysis

Data obtained from spirometry assessments were entered into a Microsoft Excel spreadsheet (Microsoft Corporation, Washington, United States) and analyzed using SPSS Statistics version 20.0 (IBM Corp. Released 2011. IBM SPSS Statistics for Windows, Version 20.0. Armonk, NY: IBM Corp.). Descriptive statistics (mean, standard deviation) were calculated for spirometric parameters. Differences in pulmonary function among subgroups were assessed using statistical tests. Student's t-tests were employed to compare means between two groups, while analysis of variance (ANOVA) was used for comparisons involving more than two groups, and a p-value of <0.05 was considered significant.

Ethical considerations

Ethical approval was obtained from the Institutional Ethics Committee (SGKH/IEC/2018/01/193) before commencing the study. Written informed consent was obtained from the parents or legal guardians of all participating children. The study was conducted in accordance with the principles outlined in the Declaration of Helsinki and local regulatory requirements. Confidentiality and privacy of participants' information were strictly maintained throughout the study.

## Results

A total of 57 children with HIV infection were included in the study, with a mean age of 13.6±3.2 years. The participants were divided into three age groups: <9 years (n=14, 24.6%), 9-11 years (n=16, 28.1%), and >11 years (n=27, 47.4%). Among the participants, 32 (56.1%) were male and 25 (43.9%) were female. The mean BMI was 15.92±2.78 kg/m2. The mean HIV viral load at the time of PFT was 87.23±56.28 copies/μL, and the mean CD4 count at PFT was 1146.32±103.98 cells/mm3. The participants had been on ART for an average duration of 6.21±1.36 years. Regarding viral load, 30 participants (52.6%) had viral loads of less than 1 copy/μL, 15 (26.3%) had viral loads between 1 and 1000 copies/μL, and 12 (21.1%) had viral loads greater than 1000 copies/μL. In terms of CD4 count categories, 27 participants (47.4%) had CD4 counts greater than 500 cells/mm3, 24 (42.1%) had CD4 counts ranging from 200 to 499 cells/mm3, and six (10.5%) had CD4 counts below 200 cells/mm3. Opportunistic infections were present in five participants (8.8%), while 52 participants (91.2%) did not exhibit any opportunistic infections (Table 1).

**Table 1:** Baseline characteristics of the pediatric HIV patients (N=57) BMI: body mass index, HIV: human immunodeficiency virus, PFT: pulmonary function testing, ART: antiretroviral therapy

Variables	Frequency	%
Mean age (in years)	13.6±3.2	
Age group		
<9 years	14	24.6
9-11 years	16	28.1
>11 years	27	47.4
Gender		
Male	32	56.1
Female	25	43.9
Mean BMI (in kg/m^2^)	15.92±2.78	
BMI		
≥18.5 kg/m^2^	21	36.8
<18.5 kg/m^2^	36	63.2
Mean HIV viral load at PFT (copies/μL)	87.23±56.28	
Mean CD4 count at PFT (cells/mm^3^)	1146.32±103.98	
Mean duration of ART	6.21±1.36	
Viral Load		
<1 copies/μL	30	52.6
1-1000 copies/μL	15	26.3
>1000 copies/μL	12	21.1
CD4 count category		
>500 cells/mm^3^	27	47.4
200-499 cells/mm^3^	24	42.1
<200 cells/mm^3^	6	10.5
Opportunistic infections		
Present	5	8.8
Absent	52	91.2

The spirometry parameters of the study participants are presented in Table 2. The mean FVC was 73.22±9.34%, while the mean FEV1 was 72.16±8.97%. The FEV1/FVC ratio, a measure of airway obstruction, was found to be 98.56%±10.41%. The PEFR exhibited a mean value of 68.51±13.74%. Furthermore, the total lung capacity (TLC) had a mean value of 77.38±16.29% (Table 2).

**Table 2:** Spirometry parameters among pediatric HIV patients (N=57) FVC: forced vital capacity, FEV1: forced expiratory volume in one second, PEFR: peak expiratory flow rate, TLC: total lung capacity

Spirometry parameters	Mean±SD
FVC (%)	73.22±9.34
FEV1 (%)	72.16±8.97
FEV1/FVC ratio (%)	98.56%±10.41%
PEFR (%)	68.51±13.74
TLC (%)	77.38±16.29

**Figure 1:**
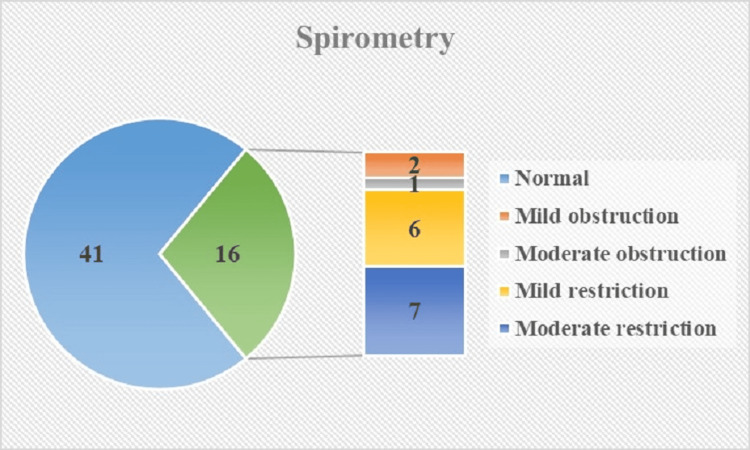
Spirometry findings among pediatric HIV patients (N=57)

Out of the total participants, 41 (71.9%) demonstrated normal spirometry results, while 16 (28.1%) had abnormal findings. Among those with abnormal results, two participants (12.5%) exhibited mild obstruction, and one participant (6.3%) showed moderate obstruction. Additionally, six participants (37.5%) had mild restriction, while seven participants (43.8%) displayed moderate restriction (Figure 1).

The spirometric parameters (mean FVC, mean FEV1, mean FEV1/FVC ratio, mean PEFR) were analyzed across different CD4 count categories: >500 cells/mm3, 200-499 cells/mm3, and <200 cells/mm3. The mean values for these parameters did not exhibit significant variations among the CD4 count categories (p>0.05 for all parameters), suggesting that pulmonary function was not significantly influenced by the CD4 count levels in the studied range. The spirometric parameters were compared based on different viral load levels: <1 copies/μL, 1-1000 copies/μL, and >1000 copies/μL. Similar to the CD4 count category, the mean spirometric values did not show significant differences among the viral load groups (p>0.05 for all parameters). This suggests that the viral load did not have a substantial impact on the measured spirometric parameters. Spirometric parameters were assessed in participants with and without opportunistic infections. The mean values of spirometric parameters did not significantly differ between the two groups (p>0.05 for all parameters), indicating that the presence of opportunistic infections did not exert a statistically significant influence on pulmonary function in this study (Table 3).

**Table 3:** Association of spirometry parameters with CD4 count, viral load, and opportunistic infections among pediatric HIV patients (N=57) * ANOVA, # unpaired t-test, HIV: human immunodeficiency virus, FVC: forced vital capacity, FEV1: forced expiratory volume in one second, PEFR: pulmonary function testing

Variables	Mean FVC (%)	Mean FEV1 (%)	Mean FEV1/FVC (%)	Mean PEFR (%)
CD4 count category*				
>500 cells/mm3 (n=27)	75.34±10.46	74.61±9.78	99.03%±9.34%	70.63±11.56
200-499 cells/mm3 (n=24)	73.82±9.57	72.62±8.82	98.37%±9.21%	69.51±12.63
<200 cells/mm3 (n=6)	72.03±8.88	70.73±8.99	98.19%±10.12%	67.51±14.22
p-value	0.72	0.575	0.961	0.842
Viral load*				
<1 copies/UL (n=30)	75.56±10.88	74.29±9.67	98.31%±8.87%	70.37±11.68
1-1000 copies/UL (n=15)	73.97±9.39	72.56±8.71	98.09%±9.27%	69.26±12.74
>1000 copies/UL (n=12)	72.12±9.19	71.37±8.73	97.85%±9.49%	67.63±14.34
p-value	0.604	0.622	0.989	0.813
Opportunistic infections#				
Present (n=5)	72.39±9.31	71.07±9.81	98.17%±10.53%	70.09±11.56
Absent (n=52)	74.75±9.89	73.58±8.99	98.43%±9.89%	67.92±14.47
p-value	0.61	0.556	0.952	0.746

## Discussion

Respiratory health is a crucial aspect of overall well-being, particularly in pediatric populations affected by chronic medical conditions. This study aimed to investigate pulmonary function using spirometry in children with HIV infection, shedding light on potential associations between various clinical parameters and lung function outcomes. The results presented in this research contribute to a broader understanding of the impact of HIV infection on pulmonary health and offer insights into the role of factors such as CD4 count category, viral load, and opportunistic infections. In our study, out of the total participants, 41 (71.9%) demonstrated normal spirometry results, while 16 (28.1%) had abnormal findings. A similar prevalence was seen in the study by Rylance et al. (25.6%) [14].

In our study, two participants (12.5%) exhibited mild obstruction, and one participant (6.3%) showed moderate obstruction. Additionally, six participants (37.5%) had mild restriction, while seven participants (43.8%) displayed moderate restriction. Earlier studies conducted by Githinji et al., Mwalukomo et al., and Rubio et al. collectively demonstrated that there were no signs of expiratory airflow limitation among individuals who were HIV positive [15-17]. Githinji et al. found that individuals with HIV infection exhibited lower measurements of flow, volume, and diffusion capacity compared to those who were uninfected [15]. Mwalukomo et al. reported an 18% prevalence of obstructive spirometry patterns and a 20% occurrence of reduced FVC in their study [16]. Masekela et al. reported a median FEV1 of 53% in their study [18].

In the study conducted by Ferrand et al., it was noted that 45% of the participants exhibited FEV1 measurements that were below 80% of the anticipated value [19]. Rylance et al. observed a comparable proportion of abnormal spirometry results between individuals who were exposed [14]. Shearer et al. found a prevalence of 21% of obstructive spirometry patterns in the HIV-infected group [11]. In the study conducted by McHugh et al., findings indicated a 10% prevalence of obstructive spirometry patterns, 18% of participants demonstrated reduced FVC, signifying potential pulmonary impairment [20].

The presence of opportunistic infections is a significant concern in children with HIV infection, and their potential impact on pulmonary function was explored in this study. Although a small subset of participants presented with opportunistic infections, the spirometric parameters did not exhibit significant differences between those with and without infections. This implies that while opportunistic infections can contribute to respiratory morbidity, as previously established, their direct influence on spirometric measurements in this cohort may be limited. A study conducted by Berman et al. revealed that 89.5% of individuals with bronchiectasis had a past history of recurrent pneumonia [21]. Weber et al. highlighted strategies such as aggressive antibiotic therapy and routine immunizations as potential approaches to managing opportunistic infections [22].

Despite the importance of CD4 count as a marker of immune health, our results indicated that varying levels of CD4 counts (>500 cells/mm3, 200-499 cells/mm3, and <200 cells/mm3) did not lead to significant differences in spirometric parameters. This implies that in children with HIV infection, pulmonary function as assessed by spirometry may not be solely determined by immune status. These findings are consistent with studies by Ferrand et al., Miller et al., and Rosen et al., indicating that although immune dysfunction is a hallmark of HIV infection, its direct effect on pulmonary mechanics may be complex and multifactorial [19,23,24]. In contrast, Nieman et al. revealed that individuals with diminished diffusing capacity for carbon monoxide exhibited a swifter progression to AIDS [25].

In our study, furthermore, the analysis of viral load and spirometric parameters revealed no significant correlations, suggesting that viral replication rates may not be a primary driver of alterations in lung function in this population. This aligns with the studies by Luzuriaga et al. and Maswabi et al., showing that the respiratory complications associated with HIV infection are often related to opportunistic infections and chronic inflammation rather than viral load levels per se [26,27]. Despite these encouraging findings, there remains a dearth of information regarding the lung function of these children.

Limitations

It is important to acknowledge certain limitations of our study. The sample size, although representative, may have influenced the ability to detect subtle associations. Additionally, the cross-sectional design limits our ability to establish causal relationships between variables and spirometric outcomes. Further longitudinal studies with larger cohorts are warranted to corroborate these findings and provide a more comprehensive understanding of pulmonary function trajectories in children with HIV infection. In addition, in India, there are still concerning rates of smoking, and understanding how environmental tobacco smoke (ETS) exposure might affect the respiratory health of HIV-infected children is a relevant area for further investigation. Future research could delve into this aspect by including questions about ETS exposure in the study questionnaire and analyzing its potential correlation with PFT results. This would provide valuable insights into the multifaceted factors influencing pulmonary function in this population and help tailor interventions accordingly.

## Conclusions

In conclusion, this study contributes valuable insights into the pulmonary function of children with HIV infection through spirometric assessments. The absence of significant associations between spirometric parameters and CD4 count category, viral load, or opportunistic infections suggests that while these factors play critical roles in the course of HIV infection, their direct impact on pulmonary mechanics might be more complex and nuanced. Continued research in this domain, with a focus on longitudinal studies and comprehensive assessments, will enhance our understanding of the interplay between HIV infection and pulmonary health, ultimately leading to improved management strategies and better outcomes for this vulnerable population.
